# The functions of repressor element 1-silencing transcription factor in models of epileptogenesis and post-ischemia

**DOI:** 10.1007/s11011-021-00719-2

**Published:** 2021-04-04

**Authors:** Ruth Butler-Ryan, Ian C. Wood

**Affiliations:** School of Biomedical Sciences, Faculty of Biological Sciences, The University of Leeds, Leeds, LS2 9JT UK

**Keywords:** Epilepsy, BDNF, Potassium channel, Transcription, Epigenetics

## Abstract

Epilepsy is a debilitating neurological disorder characterised by recurrent seizures for which 30% of patients are refractory to current treatments. The genetic and molecular aetiologies behind epilepsy are under investigation with the goal of developing new epilepsy medications. The transcriptional repressor REST (Repressor Element 1-Silencing Transcription factor) is a focus of interest as it is consistently upregulated in epilepsy patients and following brain insult in animal models of epilepsy and ischemia. This review analyses data from different epilepsy models and discusses the contribution of REST to epileptogenesis. We propose that in healthy brains REST acts in a protective manner to homeostatically downregulate increases in excitability, to protect against seizure through downregulation of BDNF (Brain-Derived Neurotrophic Factor) and its receptor, TrkB (Tropomyosin receptor kinase B). However, in epilepsy patients and post-seizure, REST may increase to a larger degree, which allows downregulation of the glutamate receptor subunit GluR2. This leads to AMPA glutamate receptors lacking GluR2 subunits, which have increased permeability to Ca^2+^, causing excitotoxicity, cell death and seizure. This concept highlights therapeutic potential of REST modulation through gene therapy in epilepsy patients.

## Introduction

Epilepsy is a prevalent neurological disorder impacting quality of life for 50 million people worldwide. Patients suffer from recurrent seizures caused by excessive firing of neurons in the brain which can often lead to hippocampal sclerosis and further deterioration. Around 30% of patients are refractory to treatment, current antiepileptic drugs often create unpleasant side effects and there are no preventative or disease-modifying epilepsy therapies. The most common type of epilepsy in adults is temporal lobe epilepsy, but acquired epilepsy is also a common result of a stroke in elderly people. Other causes are traumatic brain injury, infection or hypoxia during birth. The process of acquiring or developing epilepsy is called epileptogenesis. During epileptogenesis, the homeostatic mechanisms which protect against hyperexcitability in healthy neurons become unbalanced as the initial insult alters gene expression patterns. This is accompanied by excitotoxicity and neuronal degeneration, aberrant mossy fibre sprouting, and astrocyte-mediated inflammation (Dingledine et al. 2014).

Various animal models of epilepsy have been developed in recent decades. While no single model recreates every aspect of all epilepsies, most replicate the majority of neuropathological traits with reasonable accuracy, and the type of model used per experiment is chosen specifically for each research question. The electrical kindling model was developed in 1967, and consists of repeated short, mild tetanic stimulations every 12 or 24 h for weeks, leading to seizures of increasing severity. In the pentylenetetrazole (PTZ) kindling model, kindling is induced by an injection of a subconvulsive dose of the GABA_A_ antagonist PTZ every 2 days for up to 43 days. Kindled rodents do not typically have spontaneous seizures, but they are very easily susceptible to triggered seizures. However, animals kindled for prolonged times consisting of hundreds of trials can exhibit spontaneous seizures (Michalakis et al. 1998). In post status-epilepticus (SE) epilepsy models, SE can be induced by chemoconvulsants or a strong electrical stimulation. Most post-SE models use kainate, a glutamate analog. One large (18 mg/kg) or a few smaller (5 mg/kg) kainate injection(s) at 30 min intervals induces SE within hours, followed by a latent period and then an epilepsy-like state where spontaneous seizures can reccur indefinitely. Post-SE models are favoured for investigations of epileptogenesis because this process occurs during the latent period in patients following an initial brain insult. One common insult in the elderly population is strokes, with post-stroke epilepsy accounting for the majority of epilepsy cases in the elderly.

Epileptogenesis alters patterns of gene expression within affected brain regions. One gene of interest which is differentially regulated in epilepsy is the transcription factor Repressor Element 1-Silencing Transcription factor (REST)/Neuron Restrictive Silencer Factor (NRSF). REST is a widespread transcriptional repressor which binds to a 21-bp Repressor Element 1 (RE1)/Neural Restrictive Silencer Element (NRSE) region on its target genes via its zinc finger domain (Chong et al. 1995; Schoenherr and Anderson 1995). REST is found at high levels in non-neuronal tissues, its down-regulation is believed to be important in neuronal differentiation but it also acts as a regulator of gene expression in mature neurons (Schoenherr et al. 1996; Palm et al. 1998; Wood et al. 2003; Ooi and Wood 2007; Ballas et al. 2005). REST protein and mRNA have been found in widespread brain regions in the adult rat, including neurons in all layers of the cerebral cortex, the dentate gyrus granule cell formation and pyramidal cell layer in the hippocampus (Palm et al. 1998). There is no indication from published data that REST expression is confined to specific subclaasess of neurons. In silico analysis highlighted up to 1892 potential RE1 sites in the human genome (R. Johnson et al. 2006; Bruce et al. 2004) and orthologues across 16 other vertebrate genomes (R. Johnson et al. 2009), most of which have REST binding validated in vivo (D.S. Johnson et al. 2007). REST represses transcription by recruiting repressor complexes via N and C-terminal domains. The N-terminal domain recruits mSin3 (Grimes et al. 2000) and histone deacetylases (HDACs) 1 and 2 (Huang et al. 1999; Naruse et al. 1999; Roopra et al. 2000). Meanwhile the C-terminal domain recruits REST corepressor 1 complex [Co-REST, (Andrés et al. 1999)], which consists of further recruitment of HDACs 1 and 2, and BRG1 which stabilises REST interactions with DNA (Ooi et al. 2006; Ooi and Wood 2007). The C-terminal region also recruits the H3K4 demethylase LSD1, and the H3K9 methylase G9a (Roopra et al. 2004; Shi et al. 2004).

In animal models, REST mRNA and protein levels are consistently upregulated following induced status-epilepticus or ischemia. This occurrence across different studies is detailed in Table 1. Such increases in REST represent the observations of epileptogenesis in humans, as REST mRNA and protein levels are overexpressed in epilepsy patients, with the level of REST protein correlating with the frequency of seizures (Navarrete-Modesto et al. 2019). Kainate-induced epileptic seizures cause increased REST mRNA levels in rat hippocampal and cortical neurons in vivo (Palm et al. 1998; Spencer et al. 2006). Kainate-induced seizures in rats causes an upregulation of REST mRNA and protein for 4–48 h with a downregulation of REST target genes (Brennan et al. 2016; McClelland et al. 2011), and REST protein peaks at 24 h after kainate injection (Carminati et al. 2019). REST mRNA and protein was upregulated 24 h after pilocarpine injection, or 24 h after kindling (Hu et al. 2011a, 2011b). Interestingly, REST protein expression was increased after kindling in rats which were resistant to kindling after 20 PTZ injections, whereas REST was unchanged from control in rats which were successfully kindled after 1, 5, 10 or 20 injections (Chmielewska et al. 2020), highlighting an association between REST increase and protection against seizures in this study. Because of its upregulation following neurological insult, REST has gained some attention from research groups trying to elucidate its contribution to the epileptogenic process.

**Table 1:** List of studies using animal models of epilepsy to assess effects of insult on REST levels, and effects of REST inhibition on epilepsy pathology. Kainate (KA), PTZ, kindling and ischemia all lead to an increase in REST mRNA and protein. This REST increase can be blocked by various methods including shRNA and siRNA knockdowns, decoy oligonucleotides and cre-loxp knockout. REST is described as anti-seizure in kindling studies, pro-seizure in post-SE epilepsy models, and pro-neurodegeneration after ischemia

Model	Effect on REST	REST inhibition	Response	Role of REST	Reference
4-AP	Increased mRNA & protein	shRNA knockout	High firing rate from 4-AP remained high	Anti-epileptic	(Pozzi et al. 2013)
Kindling	Increased mRNA & protein	Cre-loxp knockout	Accelerated seizure progression	Anti-epileptic	(Hu et al. 2011a)
PTZ kindling	Increased protein in kindling-resistant rats	–	–	Anti-epileptic	(Chmielewska et al. 2020)
PTZ	–	Creloxp knockout	More PTZ required to initiate seizure	Pro-epileptic	(M. Liu et al. 2012)
Kainate	Increased mRNA	–	–	–	(Palm et al. 1998)
Kainate	Increased mRNA	–	–	–	(Spencer et al. 2006)
Kainate	Increased mRNA & protein	–	–	–	(Brennan et al. 2016)
Kainate	Increased protein	Decoy oligonucleotides	Prevented kainate-induced increase in firing rate	Pro-epileptic	(McClelland et al. 2011)
Kainate	Increased protein	Viral knockdown	Reduced & less severe seizures	Pro-epileptic	(Carminati et al. 2019)
Ischemia	Increased mRNA & protein	Antisense oligonucleotide	Rescued post-ischemic neurons	Pro-neurodegeneration	(Calderone et al. 2003)
Ischemia	Increased mRNA &protein	RNAi or dominant negative knockdown	Rescued hippocampal neurons	Pro-neurodegeneration	(Noh et al. 2012)
Ischemia	Increased protein	Pyrvinium pamoate	Rescued neurons	Pro-neurodegeneration	(Kaneko et al. 2014)
Ischemia	Increased protein	–	–	–	(Hwang et al. 2014)
Ischemia	Increased protein	siRNA knockdown	Reduced apoptosis & improved functional recovery	Pro-neurodegeneration	(Morris-Blanco et al. 2019)
Ischemia	Increased protein	Valproic acid	Protected against oxygen-glucose deprivation (OGD)-induced cell damage	Pro-neurodegeneration	(Luo et al. 2020)

## Effects of REST in post status-epilepticus seizure models

Status epilepticus (SE) in animals can be induced by a high dose injection, or a few lower dose injections of a chemoconvulsant, or a high frequency train of electrical stimulation. After the induced SE is a latent period leading to the development of spontaneous seizures. The most commonly used inducer of SE is kainate; a glutamate analog, and agonist of kainate receptors. Seizures and hippocampal damage from kainate injection was first described by Ben-Ari and colleagues (Ben-Ari et al. 1978). Kainate can induce status epilepticus following injection into the amygdala, hippocampus or ventricles. If injected into the hippocampus or amygdala, status epilepticus begins at the injection site and spreads to the ipsilateral and contralateral hippocampus, amygdala and cortex, inducing seizures within hours. This can cause neuronal loss and astrogliosis at the injection site which propogates to the hippocampus and amygdala, beginning with the hippocampal CA3 (Ben-Ari et al. 1980). Kainate has been observed causing excitotoxicity, hippocampal sclerosis and mossy fiber sprouting (de Montigny and Tardif 1981; Leite et al. 1996), as well as increasing blood brain barrier permeability. In rats with severe but not mild kainate-induced limbic seizures, blood brain barrier permeability was increased throughout the brain 2–24 h after kainate injection, before mostly returning to normal (Zucker et al. 1983). After a latency period rats then develop spontaneous recurrent seizures (Williams et al. 2009), which continue for life. A bulk of research on the functions of REST in epileptogenesis has been done using post-SE models such as kainate or the cholinomimetic pilocarpine. Here we will introduce what has been discovered about the mechanism of REST upregulation following SE and some post-SE REST targets.

### How is REST upregulated following neuronal insult?

Sirt1 is a NAD+ sensitive histone deacetylase which responds to changes in cellular energy demand by changing chromatin state and gene transcription. Activity of Sirt1 is increased in epilepsy patients and animal epilepsy models (Chen et al. 2013), suggesting that it could be involved in epileptogenesis. In 2016 the Baram group uncovered more details about how seizure causes REST to increase and found the process to be Sirt1 dependent. Kainate-induced SE in rats led to reduced microRNA-124 (miR-124) via histone deacetylation by Sirt1. The reduction in miR-124 allowed an upregulation of REST via C/EBPα increase, which had a functional effect on REST target genes, whose expression was decreased. Infusion of miR-124 agomirs also prevented REST upregulation and rescued REST target genes, though it increased inflammation and did not reduce seizures (Brennan et al. 2016). In contrast, after observing a similar downregulation of miR-124 in rats post-seizure, the Luo group administered miR-124, which alleviated PTZ and pilocarpine-induced seizures. miR-124 injection targeted and repressed cAMP-response element-binding protein 1 (CREB1), a known epileptogenesis regulator (Wang et al. 2016), highlighting CREB1 as an additional miR-124-involving pathway in seizure control that is REST-independent. miR-124 has been suggested to have a dual effect in chemoconvulsant models of attenuating seizures by inhibiting expression of REST, CREB, C/EBPα and Bcl2L13 to inhibit apoptosis and neuronal excitability and increase latency period of epileptogenesis (Schouten et al. 2015; Brennan et al. 2016; Wang et al. 2016), while promoting seizures through increased inflammation (X. Liu et al. 2019) in a REST-independent manner. In support of this, the Baram group saw no overall effect on seizures, inflammation or cell loss after rescuing post-seizure miR-124 downregulation with a Sirt1 inhibitor EX-572 following kainate-induced status epilepticus (Hall et al. 2017). Interestingly, REST and Sirt1 are also implicated in the pathology of Huntington’s disease, with increased expression of Sirt1 found in Huntington’s disease-affected post-mortem brains (Baldo et al. 2019). Huntington’s disease is associated with low levels of the REST target BDNF (brain derived neurotrophic factor), and restoration of BDNF can effectively reverse the Huntington’s disease phenotype (Xie et al. 2010), suggesting Sirt1 and REST may exacerbate the disease by downregulation of BDNF.

The REST transporter protein PRICKLE/RILP may play a role in nuclear-cytoplasmic localization and transport of REST, and mutations in PRICKLE/RILP which disturb its function lead to Progressive Myoclonus Epilepsy (Bassuk et al. 2008; Shimojo 2006). PRICKLE/RILP is also a component of the wnt signalling pathway which is well known to regulate REST expression directly (Nishihara et al. 2003). Wnt signalling pathway dysregulation is consistently observed in epilepsy and associated with aberrant neurogenesis and cell death (Hodges and Lugo 2018). Furthermore, antagonising the wnt inhibitor Dickkopf-1 has been found to reduce kainate- and ischemia-induced excitotoxicity, neuronal death and hippocampal sclerosis (Busceti et al. 2007; Cappuccio et al. 2005). Therefore it will be useful to investigate whether the REST upregulation observed in epileptogenesis is regulated by changes in the wnt pathway.

#### REST represses HCN1 channels, dopamine receptor type 2, and choline acetyltransferase

Following SE, REST has been shown to repress certain genes involved in neuronal excitation and inhibition, and understanding these effects may help to elucidate its overall role in the epileptogenic process. For example, REST binds to potassium/sodium hyperpolarization-activated cyclic nucleotide-gated channel 1 (HCN1) channels (D.S. Johnson et al. 2007). HCN1 channels contribute to control of excitability in pyramidal cell dendrites in the hippocampus and entorhinal cortex by attenuating excitatory input, and mice lacking HCN1 channels are susceptible to epilepsy (Santoro et al. 2010). Kainate-induced SE in rats caused increased REST protein and increased binding of REST to HCN1. Kainate caused an increase in number of interictal bursts and cumulative time spent in interictal activity, but this was prevented in rats injected with REST decoy oligodeoxynucleotides at 1 or 2 days after kainate-induced SE, which blocked native REST from binding and repressing HCN1. (McClelland et al. 2011). This demonstrates a pro-convulsive role for REST in the kainate model, through repression of the protective HCN1 channels.

REST may also play a pro-epileptic role through its repression of the dopamine receptor type 2 (DR2), with REST overexpression causing reduced transcript expression of the receptor (L. Lu et al. 2018), and increased DR2 mRNA levels in the striatum of REST conditional knockout mice (Yu et al. 2013). Dopamine is understood to be involved in epileptogenesis, with dopamine receptor types 1 and 2 having opposing roles. Signalling through dopamine receptor type 1 (DR1) is pro-epileptogenic while signalling through DR2 is anti-epileptogenic (Bozzi and Borrelli 2013). DR2 knockout mice have increased susceptibility to kainate and pilocarpine-induced seizures and hippocampal cell death (Bozzi and Borrelli 2002; Bozzi et al. 2000). Interestingly, DR2-like binding sites were reduced in the caudate-putamen of pilocarpine-treated rats (Yakushev et al. 2010), suggesting DR2 downregulation could be a factor contributing to the perpetuation of seizure in epileptogenesis. As REST levels increase after seizure, this DR2 downregulation could be the result of REST repression.

Another REST target gene of interest is choline acetyltransferase (Wood et al. 2003; Lönnerberg et al. 1996) which catalyses the synthesis of acetylcholine. Cholinergic dysfunction has been implicated in key mechanisms underlying temporal lobe epilepsy, though the effects of ChAT depletion are unclear (Friedman et al. 2007). Overexpression of REST caused repression of ChAT in cholinergic neurons (Lönnerberg et al. 1996), which could reflect an effect of the upregulated REST levels seen in post-SE animal models. In support of this idea, reduced ChAT activity after kainate injection was observed within hours, and also 6 months later which correlated to seizure severity (Baran et al. 2004; Guevara et al. 2002). If REST represses ChAT after seizure this could contribute to cholinergic dysfunction and epileptogenesis. More research will help to elucidate this mechanism in vivo. In contrast to the kainate model, no changes in ChAT activity were observed after kindling (Walker et al. 1985).

The idea of a pro-epileptic role for REST in post-SE models is supported by other groups. Carminati and colleagues developed a virally-delivered genetic switch for inhibiting REST in hippocampal neurons in mice. Mice expressing the REST inhibitor showed less seizure activity in response to kainate than control mice. Notably, REST inhibition also allowed the de-repression of a selection of REST target genes including potassium channels (Hcn1, Hcn3, Kcnc2, Kcnh1, Kcnip2, Kcnq1), synaptic genes (Gphn, L1cam, Nrx1, Shank, Snap25, Syn 1, Syp and several Syt) and genes involved in control of excitatory and inhibitory currents (Gabra1, Gabrg2, Glra3, Sst, Gria2, Grin1, Grin2b) (Carminati et al. 2019). Similarly, in a PTZ seizure model, threshold doses of PTZ that induced tonic-clonic seizures, and median lethal doses, were higher in conditional REST neuronal knockout mice than control mice. This demonstrates that REST knockout mice are more resistant to PTZ-induced seizures (M. Liu et al. 2012), suggesting REST incurs an overall susceptibility to PTZ, similar to kainate.

## Effects of REST after kindling

Kindling is a process of inducing an epileptic state in animal models by applying short repeated sub-convulsive stimulations, which can be either electrical or using chemoconvulsive drugs. Over time this sensitises the brain to the stimulus and creates a state of increasing excitability, beginning with brief focal afterdischarges and culminating in full generalised seizures (Sato et al. 1990). The electrical kindling animal model of epilepsy was developed by Goddard (Goddard 1967). It consists of repeated short, mild tetanic stimulations every 12 or 24 h for around 15 days inducing seizures of increasing severity. In classic electrical kindling, seizures are short and cell death and astrogliosis are not usually observed. Despite the lack of noticeable neuronal loss or synaptic terminal degeneration, aberrant sprouting of mossy fibers in the dentate gyrus has been reported from electrical kindling (Sutula et al. 1988). Notably, ‘overkindling’ caused by very prolonged kindling procedures can lead to the occurrence of spontaneous seizures (Pinel and Rovner 1978), along with some cell death and hippocampal sclerosis observed after 30 kindled seizures (Cavazos and Sutula 1990). Different brain regions have been electrically kindled, with the pyriform cortex and amygdala requiring the least stimulations, and the hippocampus and septal area requiring more stimulations to reach a kindled state (Sato et al. 1990). NMDA receptor antagonists slowed electrical kindling, suggesting their involvement in the process (Cain et al. 1988). Kindling is proposed to be reflective of epileptogenesis being driven in a controlled manner (McIntyre et al. 2002), with early seizure observations in animal models resembling complex partial seizures in the clinic, and fully kindled animal’s behaviour resembling generalised limbic seizures (Sato et al. 1990). Furthermore, specific seizure types have been recreated through electrical kindling, and antiepileptic drugs have been observed to give similar relief to each seizure type in the kindled animal models as observed in the clinic (Albright and Burnham 1980).

Chemical kindling is achieved with chemoconvulsive drugs. In this review we will focus on PTZ as it is the most commonly used drug in chemical kindling, and reflects the model used in research on the role of REST in epilepsy. PTZ kindling was first discovered by Mason and Cooper (Mason and Cooper 1972) and was described as being similar to the electrical kindling model proposed by Goddard (Goddard 1967). In PTZ kindling, repeated subthreshold doses of 20-50 mg/kg PTZ are injected into the peritoneum (Yonekawa et al. 1980). PTZ quickly distributes through the body and into the brain where it acts as an antagonist of GABA_A_ receptors, causing seizures of increasing severity and duration. PTZ kindling protocols tend to be one injection every 2 days for up to 43 days (Kanzler et al. 2018). PTZ kindling models are often used for pharmaceutical screenings but its mechanism of action is not well understood. Increased number of NMDA receptors in rat hippocampus were observed after PTZ kindling (Cremer et al. 2009), mirroring what is seen in TLE patients, and suggesting PTZ-kindling-induced epileptogenesis may be caused by increased sensitivity to glutamate (Brines et al. 1997). Mossy fibre sprouting is observed in the CA3 region of the hippocampus but not in the molecular layer of the dentate gyrus. Astrogliosis and oxidative stress are observed, alongside blood brain barrier permeability and upregulated BDNF (Uzüm et al. 2006; Zhu et al. 2015). Presence of neuronal death after PTZ kindling seems to vary by study (Tian et al. 2009; Zhu et al. 2015), and altered morphology of neurons has been described (Vasil'ev et al. 2013).

Both electrical and chemical kindling utilise the same principle of repeated subthreshold stimulations causing neuronal sensitisation to provoked seizure. One noteworthy difference between the two is that chemoconvulsants diffuse throughout the entire brain while electrical kindling begins in the region of the brain the electrodes are implanted into. This allows targeting of specific brain regions with electrical kindling, making it highly appropriate for studies involving focal seizures, whereas chemoconvulsant injection into the peritoneum are most appropriate for generalised seizures. However, in the affected brain regions the mechanisms appear similar in electrical and chemical kindling. Neuronal death has been reported in some PTZ kindling studies, and in a small percentage of electrically kindled animals, with likelihood increasing the longer the kindling procedure continues for (Zhu et al. 2015; Cavazos and Sutula 1990). The potential for neuronal damage, induction of status epilepticus and development of a state of spontaneous seizures suggests that overkindling, from either chemical or electrical stimulations, may share similarities in mechanism and effects with the post-SE models (Pinel and Rovner 1978).

### REST downregulates BDNF and TrkB in epilepsy

Brain-Derived Neurotrophic Factor (BDNF) is an established binding target of REST (Timmusk et al. 1999), and REST represses BDNF following seizures in all types of animal models examined. Inhibition of REST by decoy oligonucleotides allowed rescue of BDNF expression (Soldati et al. 2011), siRNA knockdown of REST caused a 60% increase in BDNF mRNA (Conforti et al. 2013), and kainate induced BDNF mRNA and protein increase after 24 h in cre-loxp REST knockout mice (Hu et al. 2011a, 2011b). A wealth of studies have been done which show that mRNA of BDNF and its receptor Tropomyosin receptor kinase B (TrkB) are upregulated in epilepsy models (Isackson et al. 1991; Nibuya et al. 1995; Binder et al. 1999a; Dinocourt et al. 2006; Hu et al. 2011a, 2011b), and have strong pro-epileptic effects. Knocking down or inhibiting BDNF and TrkB reduces seizures in vivo (Kokaia et al. 1995; Binder et al. 1999b; Lähteinen et al. 2002; Kang et al. 2015), while overexpression of BDNF produces mixed results (Croll et al. 1999; Scharfman et al. 2002; Eftekhari et al. 2016). It is suspected that while BDNF is pro-convulsive, it can lead to a more chronic dampening of excitation via activation of neuropeptide Y, desensitisation of TrkB and alterations in chloride conductances, evidence of which is discussed in more detail in the review of BDNF in epilepsy by Iugheti and colleagues (Iughetti et al. 2018).

Earlier this year the Szyndler group looked to investigate the links between REST and BDNF in a model of rat kindling with PTZ. The rats most resistant to kindling had significantly more REST protein than rats less resistant to kindling, but slightly less mRNA, which could have been due to an increase in protein synthesis or a decrease in degradation. Increases in BDNF were seen in kindling-resistant rats compared to the rats in kindled groups combined. TrkB protein increased in kindled rats but was not changed in kindling-resistant rats (Chmielewska et al. 2020). Such observations are consistent with the idea that increased REST repressed TrkB upregulation and protected against epileptogenesis.

Phospholipase C gamma1 (PLCγ) has been implicated as an important driver of epileptogenesis and is activated by the BDNF TrkB pathway in pilocarpine and kindling mouse models (He et al. 2010). Uncoupling of TrkB from PLCγ following SE prevented temporal lobe epilepsy (Gu et al. 2015). TrkB may induce hyperexcitability by recruiting PLCγ to help reduce expression of the K-Cl cotransporter KCC2, resulting in a reduction of Cl^−^ dependent hyperpolarizing postsynaptic current (Rivera et al. 2004). Interestingly, KCC2 is directly regulated by REST in development, when it is involved in the GABAergic switch from excitatory to inhibitory neurotransmission (Yeo et al. 2009), so its RE1 sites might allow REST to contribute to its repression in adult epileptogenesis. All the evidence suggests the TrkB increase after seizure to be proconvulsant, and inhibition of TrkB activity by REST repression of BDNF in the kindling model reduces epilepogenesis.

### REST may contribute to the antiepileptogenic effects of the ketogenic diet

The ketogenic diet is a high fat, very low carbohydrate diet which is used to control seizures in people with drug-resistant epilepsy. Its mechanisms of action are not fully understood but likely involve multiple pathways inducing antiseizure and antiepileptogenic effects. Investigation of the role of glycolysis in epilepsy has made use of 2-Deoxyglucose (2DG) to inhibit glycolysis as a mimic for the ketogenic diet. In 2006 the Roopra group showed that 2DG application blocked epileptogenesis in a rat electrical kindling model and prevented post-seizure upregulation of BDNF and TrkB mRNA, through enhanced repression by REST and the REST co-repressor C-terminal-Binding Protein 1 (CtBP). Glycolytic inhibition enhanced REST recruitment of the NADH-sensitive CtBP. This interaction was disrupted by increasing NADH levels as would occur during glycolysis, suggesting glycolytic inhibition allows REST to bind CtBP more strongly through a reduction of NADH and provide an enhanced level of gene repression (Garriga-Canut et al. 2006). The requirement of REST for 2DG-mediated prevention of electrical kindling was confirmed by the Xiong group, as REST conditional knockout mice were resistant to the antiepileptic effect of 2DG. However, the ketogenic diet blocked electrical kindling in both control and REST knockout mice, demonstrating that REST is not required for the antiepileptic effects of the ketogenic diet (Hu et al. 2011a, 2011b). The ketogenic diet has been shown to exert its antiepileptic effects through a few different mechanisms (Boison 2017), but the results mentioned here suggest REST repression of BDNF to be a contributory factor.

## Effects of REST in homeostasis after 4-AP

Homeostatic plasticity is a regulatory adjustment in the strength of neuronal firing in response to sustained excitatory changes, and can be in the form of intrinsic excitability or synaptic scaling. Work by the Baldelli group has implicated REST in both types of plasticity in response to heightened excitation by the Kv1 voltage gated K^+^ channel blocker and convulsant 4-aminopyridine (4-AP) (Pecoraro-Bisogni et al. 2018; Pozzi et al. 2013). REST was found to protect against 4-AP-induced hyperexcitability through downregulation of voltage-gated sodium channels. Cultured hippocampal neurons were transfected with REST, which reduced 4-AP induced neuronal excitability and sodium current density, as well as downregulating expression of voltage-gated sodium channels. Scrambled shRNA 4AP-treated neurons had a high initial firing rate which then reduced by 48 h, but in REST shRNA knockout neurons, 4-AP induced a high firing rate which remained high, suggesting REST was necessary for the reduction in network excitability (Pozzi et al. 2013). The group also later showed that 4-AP induced prolonged hyperexcitability and REST increase which decreased the size of the synaptic vesicle pools. This groups work demonstrates how synaptic scaling and intrinsic homeostatic plasticity are both regulated by REST, to prevent hyperexcitability (Pecoraro-Bisogni et al. 2018). Similar to in the in vivo kindling models of epileptogenesis, acute in vitro 4-AP seizure models increase neuronal excitability without most of the excitotoxicity and cell death observed in post-SE models. In accordance with kindling models, 4-AP models portray REST as protective against hyperexcitability.

## Effects of REST after ischemia

Epilepsy can be acquired in response to a stroke, traumatic brain injury, tumour or infection. Most strokes are ischemic, and ischemic injury leads to excitotoxicity, inflammation, neuron and astrocyte death, mitochondrian dysfunction and free radical production (George and Steinberg 2015). Following ischemia, microglia and astrocytes are activated which trigger an inflammatory response by upregulation of NF-kB (W. Liu et al. 2011). Hypoxia from hypoperfusion quickly causes brain damage. Large infarctions are associated with recurrent post-stroke seizures (Gupta et al. 1988). Around 14% of surviving ischemic stroke patients develop seizures (Kotila and Waltimo 1992), referred to as post-stroke epilepsy as the seizures are spontaneous and repetitive. The risk of post-stroke seizures is associated with the severity of the stroke and resulting brain damage, disability after stroke and hippocampal involvement (Myint et al. 2006). Post-stroke epilepsy seems to be generated by hypoxia and metabolic dysfunction, breakdown of the blood brain barrier, glutamate excitotoxicity and the loss of neurovascular unit integrity (Reddy et al. 2017). Animal ischemic stroke models can be global or focal. Global cerebral ischemia is induced by 2 or 4 vessel occlusion. Focal cerebral ischemia is transient and is usually induced by middle cerebral artery occlusion (MCAO). Twelve months after MCAO in primates, there were fewer neurons and more glia in the hippocampus than in control groups, but the infarct was restricted to cortical regions (Ouyang et al. 2020). In summary, the ischemic model induces some similar physical pathologies as the post-SE models, focussing heavily on neuron death, excitotoxicity, inflammation and infarction, whilst usually lacking the neuronal hyperexcitability observed in the post SE and kindling models.

In ischemia models REST increases consistently exacerbate cellular damage and may drive epileptogenesis. Following ischemic insult, REST increase was inhibited by injection of the casein kinase 1 (CK1) activator pyrvinium pamoate. CK1 phosphorylates REST, targeting it for proteasomal degradation. As well as blocking REST increase, CK1 activation also rescued neurons from cell death (Kaneko et al. 2014). The same year, the group identified miR-132 as a target of REST in ischemia. miR-132 is important for synaptic plasticity and synaptogenesis. REST depletion inhibited ischemia-induced repression of miR-132 by REST. Moreover, overexpression of miR-132 in vivo by lentiviral delivery conferred protection of neurons against ischemia-induced cell death (Hwang et al. 2014). REST upregulation after MCAO-induced ischemia-reperfusion was also associated with a decrease in HCN1. This was rescued by valproic acid, which also protected neurons from damage caused by oxygen-glucose deprivation and reoxygenation (Luo et al. 2020). Another mechanism REST uses to amplify neurodegeneration is through repression of the anti-excitatory AMPA receptor subunit GluR2 (Huang et al. 1999). GluR2 has anti-excitatory effects through restriction of Ca^2+^, and GluR2 knockdown in animal models is associated with enhanced epileptogenesis by increasing the permeability of AMPA receptors to Ca^2+^ ions (Sanchez et al. 2001). REST mRNA and protein increased after ischemia causing repression of GluR2 alongside Grin1 and other genes, while REST knockdown prevented GluR2 repression and rescued neurons from ischemia-induced death of hippocampal neurons in vivo (Calderone et al. 2003; Noh et al. 2012). GluR2 is also reduced after SE (Grooms et al. 2000). After MCAO-induced REST increase, REST siRNA knockdown caused considerable upregulations in GluR2 and Grin1, as well as BDNF. REST knockdown also improved functional recovery, apoptosis and infarct volume (Morris-Blanco et al. 2019). REST repression of GluR2 and Grin1 after ischemic insult promotes brain damage (Mehta et al. 2015). It is hypothesised that Ca^2+^ permeable AMPA receptors may cause excitotoxic cell death to interneurons, which increases overall hyperexcitability by reducing inhibitory GABA signalling (Rogawski and Donevan 1999). In contrast, REST has also been shown to repress expression of the MOR-1 (mu opioid receptor 1) within inhibitory interneurons in the CA1 hippocampal region following ischemic insult which may be neuroprotective. Knocking down MOR-1 through antisense oligonucleotides or the MOR-1 antagonist naloxone conferred protection of pyramidal neurons to ischemia-induced cell death, potentially by disinhibiting GABA release from inhibitory interneurons, demonstrating MOR-1 to be pathological in this model (Formisano et al. 2007). Despite REST’s protective potential with MOR-1, research clearly indicates REST to drive neuron death in ischemic models, through GluR2.

In addition to ischemia, REST’s involvement in cell death occurring from other types of insult seems likely. Methylmercury has been shown to cause neurotoxicity through increased expression of REST and deacetylation of histone protein H4 in SH-SY5Y cells, and in the mouse cerebellar granule cell layer. The methylmercury-induced neurotoxicity in SH-SY5Y cells was prevented by the HDAC inhibitor trichostatin-A through prevention of H4 deacetylation (Guida et al. 2016). REST has also been shown to increase after inflammation in vitro in dorsal root ganglia neurons, which repressed expression of Kv7 channel subunits KCNQ2 and KCNQ3, and reduced the Kv7 current. Kv7 channels are anti-excitatory, producing a subthreshold potassium current which stabilises the resting membrane potential, so their repression by REST would be expected to increase excitability (Mucha et al. 2010). Mutations in the subunits composing Kv7 channels are associated with the neonatal epilepsy Benign Familial Neonatal Convulsions, and the antiepileptic drug Retigabine works by opening Kv7 channels. This mechanism was characterised further for its role in chronic pain (Rose et al. 2011; Zhang et al. 2019). Further research will determine whether a parallel mechanism of increased REST downregulating anti-excitatory Kv7 channels in the CNS could drive epileptogenesis following ischemia or SE.

## Why does REST show different function in different models?

**Fig. 1:**
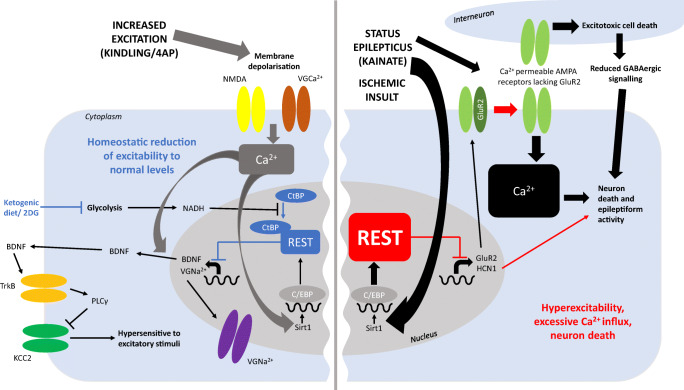
The differential contributions of REST in seizure and ischemia models in pyramidal neurons. REST has either an overall protective (blue) or pathological (red) contribution to the effects of kindling and 4-AP seizure models (left), or status epilepticus seizure models and ischemia models (right), respectively. An excitatory stimulus within the ‘normal’ range for neurons, such as kindling in vivo, or 4-AP in vitro, causes membrane depolarization and Ca^2+^ influx through NMDA receptors and voltage gated Ca^2+^ channels. The Ca^2+^ increase enhances expression of BDNF and increases activity of Sirt1. This leads to an increase in the transcription factor C/EBP which enhances expression of REST. REST represses expression of voltage gated Na^2+^ channels and BDNF. BDNF protein is secreted outside of the cell, where it binds to TrkB receptors, activating PLCγ. PLCγ recruitment by TrkB represses expression of KCC2 transporters, leading the cell to a state of hypersensitivity of cells to excitatory stimuli. This process is downregulated by REST-mediated repression, as is the expression of voltage gated Na^2+^ channels which contribute to action potential spiking. Therefore the actions of upregulated REST in this system lead to an overall homeostatic reduction of excitability towards normal levels. In contrast, status epilepticus or administration of chemoconvulsants such as kainate cause a greater excessive influx of Ca^2+^ to the cell, leading to a larger increase in REST levels. In addition to the downstream effects seen in normal levels of excitation (left of diagram), REST here is also able to repress HCN1 and the AMPA receptor subunit GluR2. The repression of GluR2 leads to an increase in AMPA receptors lacking the GluR2 subunit, which have increased permeability to Ca^2+^. This allows an excessively large Ca^2+^ influx, which is also triggered by status epilepticus. The excitotoxic death of nearby interneurons induced by Ca^2+^ influx leads to reduced GABAergic signalling to the pyramidal neuron. In combination with the excessive Ca^2+^ influx, this reduced inhibition is sufficient to trigger neuron death in ischemia models, and additional epileptiform activity in chemoconvulsant seizure models. Repression of HCN1 expression by REST also contributes to the epileptiform activity and neuron death triggered by kainate exposure

This review describes the research which has investigated the function of the REST upregulation seen following a brain insult which could provoke seizures. Table 1 shows the commonality of REST mRNA and protein increasing consistently in all studies and models. REST regulates many different genes which can individually contribute pro- or anti-excitatory effects (see Fig. 1), and the balance between all of these factors may determine whether or not an individual goes on to develop epilepsy following an initial brain insult.

SE in animal models can be induced by an array of chemoconvulsants including pilocarpine, picrotoxin, bicuculline, kainate, or PTZ. Post-SE models have been described as more accurate than kindling models in creating all the features of mesial temporal lobe epilepsy (TLE). The latent period is particularly relevant for the process of epileptogenesis, and the mossy fibre sprouting and hippocampal sclerosis are common pathological hallmarks of TLE. SE leads to an increase in mossy fiber sprouting, neuron death, and increases in NMDA and AMPA receptor expression, compared to kindled rats (Mathern et al. 1997). The presence of any harmful secondary effects from kindling does seem to depend upon the length of the kindling procedure. Classical electrical kindling and most PTZ kindling procedures do not induce spontaneous seizures or cause significant neuronal damage, but more severe secondary effects are observed with longer kindling protocols (Pinel and Rovner 1978). Kindling is criticised as only being a partial model of epilepsy, recapitulating hyperexcitability without neuronal damage, and is being used less commonly compared to post-SE models in recent years. The main drawback of post-SE models is that the degree of cell death and hippocampal sclerosis can be much more pronounced than in epilepsy patients. The brain damage in animal models occurs from the initial SE (Gorter et al. 2003). In contrast to pure epilepsy models, ischemia reliably models the effects of stroke, though only 14% of ischemic strokes lead to epilepsy (Kotila and Waltimo 1992).

In the kindling model REST is anti-excitatory, whereas in models with an insult that induces cell death, REST seems to augment the cell death and evoke seizures (See Table 1). This difference may be facilitated by the level of REST increase induced. In the PTZ kindling model, Chmielewska and colleagues reported REST protein to be around 650 pg/mg in kindling-resistant rats compared to around 500 pg/mg in successfully kindled rats and unkindled control rats (Chmielewska et al. 2020). In contrast, kainate-induced SE typically causes REST protein levels to increase 2–4 fold within 48 h (McClelland et al. 2011; Carminati et al. 2019; Brennan et al. 2016). Similarly, REST protein is consistently seen to increase 2–3-fold in the CA1 hippocampal region and the peri-infarct cortical tissue at 12–48 h after ischemic insult, though no significant changes are observed in the CA3 region or the dentate gyrus (Calderone et al. 2003; Kaneko et al. 2014; Hwang et al. 2014; Morris-Blanco et al. 2019). Therefore, small increases in REST protein may allow a homeostatic downregulation of excitability in response to increased neuronal activity, but large increases in REST may lead it to adopt a more pathological role. Interestingly, exposure of hippocampal neurons to 4-AP in vitro for 96 h led to a 7-fold increase in REST, which was reported to be anti-excitatory (Pozzi et al. 2013). This could be because the in vitro single cell nature of the experimental design did not have the necessary factors to allow REST to become pro-seizure. For example, REST expression by glial cells may also be necessary for its pro-seizure effects. This suggests the level of REST protein is not the only factor determining the role of REST in epilepsy.

Kindling seems to depend on the upregulation of BDNF and TrkB, with deletion of BDNF and TrkB reducing or abolishing kindling effects, respectively (Kokaia et al. 1995; He et al. 2004). TrkB contributes to the development of kindling, and its levels are increased in successfully kindled rats, but not kindling-resistant rats (Binder et al. 1999a; Chmielewska et al. 2020). Kindling-resistant mice also had increased recruitment of CtBP by REST at the BDNF promoter (Potter et al. 2010). In normal physiological fluctuations in excitability, REST likely acts as a brake by repressing BDNF and TrkB, allowing excitability levels to return to normal. Successful kindling provokes this homeostatic mechanism but the modest REST increase involved is not great enough to completely prevent an increase in BDNF and TrkB, so a kindled state develops. There is other evidence of REST acting in a neuroprotective manner in healthy brains. REST levels rise in healthy brains during ageing, preventing oxidative stress and repressing genes involved in the promotion of Alzheimer’s disease (T. Lu et al. 2014). During ageing neuronal excitation increases. REST represses genes involved in excitation, with reduced excitation associated with increased lifespan (Zullo et al. 2019). The combined evidence suggests that in healthy brains, REST upregulation allows a protective reduction in neuronal excitability, protection against oxidative stress and increasing longevity.

SE and ischemic insults appear to cause a larger increase in REST than kindling, and this additional REST may cause it to impact additional pathways. GluR2 protein is decreased by around 40% in CA1 and CA3 pyramidal neurons 24 h after kainate-induced SE, which is associated with neuronal death (Grooms et al. 2000). Ischemic insult leads to a reduction in GluR2 protein of over 40% in CA1 after 48 h (Calderone et al. 2003; Noh et al. 2012). In contrast, after kindling, no reduction of GluR2 protein is observed in the hippocampus at all, though a 20–30% reduction is seen in the piriform cortex/amygdala and limbic forebrain regions (Prince et al. 1995). This may suggest a correlation between increasing insult severity and level of hippocampal REST increase, and GluR2 downregulation in the hippocampus, with the piriform cortex/amygdala and limbic forebrain being affected by GluR2-mediated damage first. Following SE or ischemia, the large REST increase causes HDAC-dependent repression of GluR2, which leads to neuron death through Ca^2+^ permeable AMPA receptor-mediated excitotoxicity. This mechanism explains why REST is pro-neurodegenerative in ischemia models. In kainate models, kainate causes seizures by activating AMPA receptors. The AMPA receptors are deficient in GluR2 due to REST downregulation of GluR2 and are more permeable to Ca^2+^, so activation by kainate furthers the AMPAR-mediated excitotoxicity, inducing seizures. Kainate-induced AMPAR-mediated currents were increased in receptors lacking GluR2 (Iihara et al. 2001). REST acts in a pro-epileptogenic fashion in the kainate model because its post-seizure increase causes the downregulation of GluR2, exacerbating AMPAR-mediated excitotoxicity. This REST-mediated cell death is likely a significant driving factor behind epileptogenesis. Glutamate-mediated excitotoxicity is the main cause of hippocampal pyramidal cell death in epilepsy, through necrosis, apoptosis, and autophagy. Excitotoxicity causes hyperactivation of glutamate receptors leading to increased calcium influx into the cell and the mitochondria, causing metabolic dysfunction, free radical generation, inhibition of protein synthesis and activation of nitric oxide synthase (NOS), proteases, phospholipases, endonucleases. Activation of NOS causes damage to the neuronal membrane and this cell damage leads to seizures (Lorigados Pedre et al. 2013). A similar effect is seen in the PTZ seizure model through attenuation of inhibitory GABA signalling (M. Liu et al. 2012). If higher levels of REST are required to induce GluR2 repression than BDNF repression, this would suggest REST may bind BDNF more strongly than GluR2. REST is known to bind the GluR2 promoter less strongly than then NaII promoter (Myers et al. 1998), but research is needed to compare REST binding to GluR2 or BDNF. In addition to GluR2, grin1 and chrnb2 are also repressed by REST following ischemia (Noh et al. 2012), and mutations in both of these are associated with seizures (Phillips et al. 2001; Lemke et al. 2016), highlighting them as more potentially interesting avenues of investigation in REST-mediated epileptogenesis.

The exacerbation of neurodegeneration by REST may be an important driver of epileptogenesis in post-stroke epilepsy and temporal lobe epilepsy with hippocampal sclerosis. It may be beneficial to investigate the function of REST in models of traumatic brain injury (TBI), as this does not appear to have been looked at. TBI is more likely than ischemic injury to result in epilepsy. Like ischemia and SE, TBI leads to excitotoxicity and cell death, inflammation and blood brain barrier dysfunction (Sande and West 2010). Therefore, like in ischemia and SE models, it seems likely that REST would be strongly upregulated following TBI, which could contribute to neuronal death and encourage seizure activity.

## Conclusion

Epileptogenesis involves a selection of cellular and molecular changes following an initial brain insult and a latent phase, which make neurons more susceptible to hyperexcitability and spontaneous seizures. However, we are far from understanding the mechanisms responsible and knowledge of such mechanisms may help identify therapeutic strategies. In addition to larger structural changes such as the loss of inhibitory interneurons and aberrant axonal sprouting, changes at the mRNA and protein levels have also been explored to uncover some molecular pathways involved in seizure progression. The abundance of data implicating REST in the process of epileptogenesis makes it a strong candidate as a master switch, triggering a cascade of downstream events to modulate excitability of each individual neuron. Sirt1 activity increases after insult, likely by sensing an increased metabolic demand in the cell, and this represses miR-124, which allows upregulation of REST (Chen et al. 2013; Brennan et al. 2016). The likely involvement of REST in the antiepileptic effects of the ketogenic diet has been demonstrated as it is activated by the glycolysis inhibitor 2DG and represses the pro-convulsant BDNF (Garriga-Canut et al. 2006). BDNF and its receptor TrkB are both upregulated in epilepsy and their repression by REST seems to be an important regulatory switch to protect against epileptogenesis (Isackson et al. 1991; Nibuya et al. 1995; Binder et al. 1999a, 1999b; Dinocourt et al. 2006). REST downregulates voltage-gated sodium channels and sizes of synaptic vesicle pools, reducing intrinsic excitation and synaptic scaling (Pozzi et al. 2013; Pecoraro-Bisogni et al. 2018), while its downregulation of GluR2 and HCN1 increases excitation (McClelland et al. 2011; Grooms et al. 2000; Sanchez et al. 2001; Huang et al. 1999). It seems likely that REST homeostatically protects healthy brains from increases in excitability through downregulation of BDNF and TrkB, but takes on a pathological role post-seizure in epileptic brains by repressing GluR2, helping to drive epileptogenesis. For epileptic patients, modulation of specific REST pathways could provide relief from seizure-induced brain damage and epileptogenesis. More defined studies should unravel specific roles of REST in particular types of seizure in the clinic and assess for the potential to use REST modulation as a therapy.

## Data Availability

Not applicable.
